# Detection of Severe Murine Typhus by Nanopore Targeted Sequencing, China

**DOI:** 10.3201/eid2906.221929

**Published:** 2023-06

**Authors:** Panpan Qian, Xiaohua He, Mei Yang, Li Wei, Lihui Zhang, Xiqian Xing

**Affiliations:** The Affiliated Hospital of Yunnan University, Kunming, China

**Keywords:** Murine typhus, Rickettsia typhi, bacteria, rickettsia, vector-borne infections, zoonoses, multiple organ dysfunction syndrome, nanopore targeted sequencing, China

## Abstract

We report a case of murine typhus in China caused by *Rickettsia typhi* and diagnosed by nanopore targeted sequencing of a bronchoalveolar lavage fluid sample. This case highlights that nanopore targeted sequencing can effectively detect clinically unexplained infections and be especially useful for detecting infections in patients without typical signs and symptoms.

Murine typhus is caused by *Rickettsia typhi* bacteria transmitted by rat or cat flea vectors. Persons with murine typhus often have nonspecific or mild symptoms, such as fever, myalgia, and rash. In rare instances, murine typhus will cause atypical or multiple organ dysfunction syndrome (MODS) (*1*,*2*). 

Murine typhus is an undifferentiated febrile illness, which makes it challenging to recognize and diagnose. We report a case of murine typhus and MODS in a patient without rash. We diagnosed murine typhus by using nanopore targeted sequencing (NTS) of a bronchoalveolar lavage fluid (BALF) sample, aiming to provide more reference for clinical practice. 

A 60-year-old female farmer from Yunnan Province, China, had fatigue, anorexia, nausea, dizziness, and vomiting for 1 week. At admission, she was afebrile and hemodynamically stable and did not have headache, rash, or eschar. Chest computed tomography (CT) imaging showed pneumonia and a small plural effusion (Figure, panels A, B). By the next day, her condition had deteriorated. She experienced chills, fever (temperature 39°C), severe hypotension (70/53 mm Hg), dyspnea, and deterioration of the oxygenation index. Preliminary laboratory investigation demonstrated mild leukocytosis (13.86 × 10^9^ cells/L), moderately elevated transaminase levels (alanine aminotransferase 197 U/L, aspartate aminotransferase 128 U/L), severe thrombocytopenia (12 × 10^9^ platelets/L), coagulation disorder (D-dimer 49.8 µg/mL), elevated C-reactive protein (207.4 mg/L) and procalcitonin (4.65 ng/mL) levels, and respiratory failure (partial pressure of oxygen 58.9 mm Hg).

**Figure:**
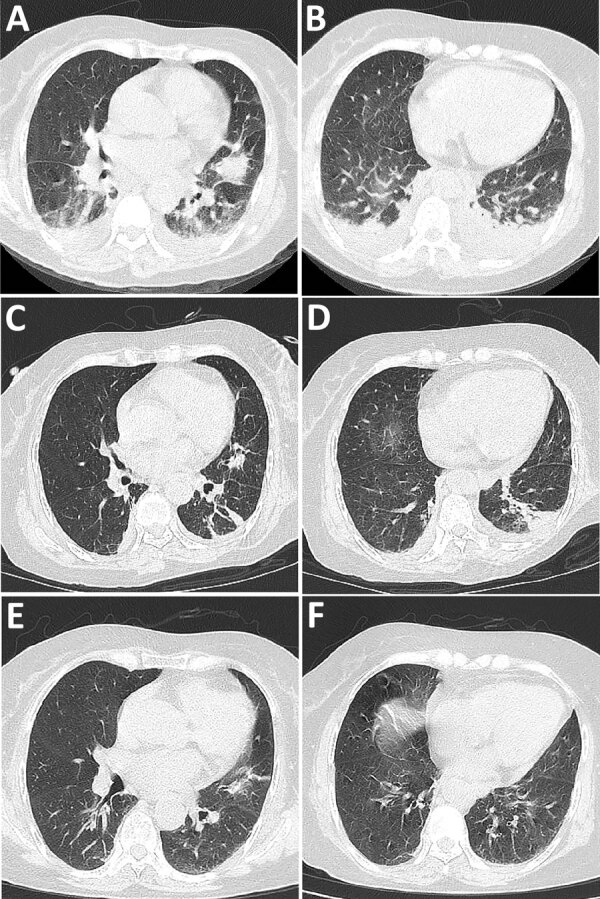
Chest computed tomography images from a patient with severe murine typhus detected by nanopore targeted sequencing, China. A, B) Images taken at hospital admission demonstrating pneumonic exudation of the left lung lingual segment and double lower lobes and small plural effusion. C, D) Improvement of pulmonary infiltrates after 14 days. E, F) Resolution of pulmonary infiltrates demonstrated 1 month after hospital discharge.

The patient was given intravenous meropenem and norepinephrine and was admitted on noninvasive ventilation. We then conducted tests for malaria, *Legionella*, influenza virus, SARS-CoV-2, HIV, herpes simplex virus, cytomegalovirus, Epstein-Barr virus IgM, *Roxiella burnettii* IgM (phase II antigen), *R. typhi* IgM, *Mycoplasma pneumoniae* IgM, *Chlamydia* IgM, respiratory syncytial virus IgM, and adenovirus IgM; results were all negative. In addition, testing of blood, urine, stool, and sputum cultures and bone marrow biopsy all produced negative results.

On admission day 5, she remained normotensive. Her body temperature dropped, but she still had a low-grade fever, body temperature fluctuating from 37.5°C to 38°C. However, the cause of her severe infection remained unclear. The next day, she underwent bronchoscopy. BALF was sent to undergo NTS analysis to Wuhan Dgensee Clinical Laboratory Co., Ltd (https://www.dgensee.com). Two days later, NTS results revealed *R. typhi* DNA.

NTS analysis yielded a total of 71,252 single-end reads. *R. typhi* had the highest relative abundance of 44.58% (n = 31,764 reads). Subsequently, we conducted quantitative PCR (qPCR) using a sequence from GenBank (accession no. WP_011190964) as the target gene to detect *R. typhi*. The qPCR results confirmed the NTS detection (Appendix).

After the murine typhus diagnosis, the patient was treated with doxycycline (100 mg every 12 h) beginning on admission day 9. Soon after, her body temperature, platelet count, and blood coagulation function returned to normal. Reexamination of chest CT images showed improved pulmonary infiltrates (Figure, panels C, D), and she was discharged from the hospital. She continued doxycycline (100 mg 2×/d) for 14 days after discharge. At a 1-month follow-up, she had no symptoms of discomfort, and chest CT imaging showed resolving pulmonary infiltrates (Figure, panels E, F).

As an undifferentiated febrile illness, murine typhus can be challenging to diagnose in clinically mild or severe illness. Murine typhus can manifest with nonspecific symptoms and mimic other disease processes, making laboratory-confirmed diagnosis difficult without a high index of suspicion.

Diagnosis of murine typhus is usually performed by serologic testing and molecular analysis. Adaptation of modern serologic techniques for murine typhus diagnosis has substantially increased diagnostic accuracy, and serology was deemed the standard before the wide acceptance of molecular testing. Rickettsial infections require early diagnosis and treatment to prevent severe outcomes, but early diagnosis is rarely achieved by using serology (*3*). In addition to serology, the most common diagnostic method for murine typhus is qPCR (*4*). However, PCR has limitations, including being more sensitive during acute illness, such as the febrile phase, usually days 1–5 of illness, but possibly up to days 7–10 (*3*).

NTS is a groundbreaking technology that has the potential to overcome the shortcomings of both PCR and metagenomic next-generation sequencing, and next-generation sequencing is much less affected by antimicrobial drugs than is PCR (*5*–*7*). NTS has been used clinically and has shown high specificity and sensitivity (*6*). NTS combines long read length (>5,000 bp) and targeted amplification of 16S RNA gene for bacteria, *rpoB* for mycobacteria, and internal transcribed spacer (ITS) for fungi, all of which are free from interference of host background DNA (*8*,*9*). NTS can accurately detect causative pathogens in infectious samples and has a short 8–14-hour turnaround time.

In conclusion, we successfully detected *R. typhi* by using NTS in a febrile patient with MODS but without rash. NTS is a promising technology that can efficiently identify infectious pathogens early and has the potential to assist physicians in providing timely and precise treatment, especially for patients with nonspecific symptoms indicative of multiple disease processes.

Appendix Additional information on detection of severe murine typhus by targeted nanopore sequencing, China.
