# Supplementary material for: Detection of Severe Murine Typhus by Nanopore Targeted Sequencing, China
Source: Emerg Infect Dis. 2023 Jun;29(6):1275–7. doi: 10.3201/eid2906.221929 (PMC10202882; doi:10.3201/eid2906.221929)
Supplement: Appendix — Additional information on detection of severe murine typhus by targeted nanopore sequencing, China. [file 22-1929-Techapp-s1.pdf]

Article DOI: https://dpoi.org/10.3201/eid2906.221929

EID cannot ensure accessibility for supplementary materials supplied by authors. Readers who have difficulty accessing supplementary content should contact the authors for assistance.

Detection of Severe Murine Typhus by Nanopore Targeted Sequencing, China

Appendix

Sample Processing and DNA Extraction

All samples were collected into sterile tubes and sent to the clinical laboratory for DNA extraction. BALF samples were centrifuged at 20,000 × g for 10 min and the supernatant was removed. Then 200 µL of the sample was retained for DNA extraction. As for blood, 1.5 mL of EDTA whole blood samples were centrifuged at 800 × g for 10 min at room temperature and the lower part of red blood cells was discarded. About 600 µL of supernatant, including leukocytes and plasma, were separated and transferred to a new Eppendorf tube. Then the tube was centrifuged at 16,000 × g for 10 min and the supernatant was discarded. About 200 µL of precipitate was collected for subsequent DNA extraction. DNA was extracted using the Sansure DNA Extraction Kit (Changsha, China) following the manufacturer's instructions. At the same time, 200 µL Tris-EDTA buffer was added in the batch as the negative control for DNA extraction (extraction control, ETC).

Amplification and Nanopore Targeted Sequencing

NTS was built by targeted amplification of the 16S rRNA gene (for bacteria), ITS1/2 gene for fungal, and rpoB for *Mycobacterium* spp. by using universal and specific primers, and sequenced by a real-time nanopore sequencing platform. The 27F/1492R and ITS1/4 primers were employed as the start primers for amplification of bacterial 16S rRNA and fungal internal

transcribed spacer regions 1 and 2 (ITS1/2), respectively; the additional primers with barcode are also listed in Appendix Table 1, which would make organisms amplified and sequenced successfully by reducing the risk of amplification failure created by variation of each base (*4–7*). All the primers should meet the following standards: (i) 18–30 bp for primer length; (ii) melting temperature (Tm): 58°C–65°C, with a temperature difference of less than 3°C between start tube and additional primer; (iii) GC content of primers: 40%–60%; (iv) ΔG (Gibbs free energy) of the last five resides of the primers at the 3′ end: ≥–9 kcal/mol (M. Wang et al., unpub. data, https://doi.org/10.1101/2020.04.08.20057604). The 27F/1492R or ITS1/4 primer and its additional primers was blended with the molar ratio of 3:1 for amplification. For mycobacterial rpoB, MF/MR primer and the additional primers were especially mixed with the molar ratio of 3:1 to obtain the final specific primer pairs (Appendix Table 1). The full lengths of 16S rRNA, ITS, and rpoB are ≈1.5 kb, 400–800 bp, and ≈400 bp in this study. Amplification of the 16S rRNA gene and rpoB were performed in a 20 µL reaction system with 8 µL extracted DNA, 2 µL barcoded primer consisting of random N bases (10 µM), and 10 µL 2× KOD OneTM PCR Master Mix (TOYOBO) using the following procedure: 1 cycle at 98°C for 3 min, 35 cycles at 98°C for 10 s, 55°C for 5 s, and 68°C for 10 s, followed by a final elongation step at 68°C for 5 min. ITS1/2 was first amplified using the same reaction system, and PCR procedure was performed using the primer for ITS1/2 without a barcode (1 cycle at 98°C for 3 min, 35 cycles at 98°C for 10 s, 55°C for 5 s, and 68°C for 10 s, followed by a final elongation step at 68°C for 5 min); the PCR product was purified using 0.8× AMpure beads (Beckman Coulter) and eluted in 10 µL Tris-EDTA buffer. Then, 5 µL of the eluate was used for the barcoded PCR with 5 µL of the barcoded ITS1/2 primer set (10 µM) and 10 µL 2× Phusion U Multiplex PCR Master Mix using the following procedure: 1 cycle at 98°C for 3 min, 10 cycles at 98°C for 10 s, 55°C for 5 s, and 68°C for 5 s, followed by a final elongation step at 68°C for 5 min. The barcoded amplification products of the 16S rRNA gene, ITS1/2, and rpoB from the same samples were pooled at a mass ratio of 10:3:1. The pooled products from the different samples were equally mixed and used to construct sequencing libraries using the 1D Ligation Kit (SQK-LSK109;

Oxford Nanopore). Clinical samples and two Tris-EDTA buffers (no-template control, NTC) were batched in one sequencing library, and the library was sequenced using Oxford Nanopore GridION X5 with real-time base calling enabled (ont-guppy-for-gridion v. 1.4.3–1 and v. 3.0.3–1; high-accuracy base calling mode).

Bioinformatics Analysis Pipeline

Base-calling and quality assessment of sequencing data were performed by using Oxford Nanopore GridION X5 and Guppy in high accuracy mode (ont-guppy-for-gridion v. 1.4.3–1 and v. 3.0.3–1; high-accuracy base calling mode). Sequencing reads with low quality (Q score <7) and undesired length (<200 nt or >2,000 nt) were discarded. An in-house script was used to analyze the output of the base calling data and generate a real-time taxonomy list of each sample by screening and starting the bioinformatic pipeline when every 4,000 reads passed the base calling process. Briefly, Porechop (v. 0.2.4) was used for adaptor trimming and barcode demultiplexing for retained reads that passed the basecalling process. The reads of each sample were mapped against the 16S rDNA/ITS reference database collected from NCBI FTP (ftp://ftp.ncbi.nlm.nih.gov/refseq/TargetedLoci) using BLAST. Reads with alignments that exhibited both >80% identity and >80% query coverage were retained (*8*). Then, the taxonomy of each read was assigned according to the taxonomic information of the mapped subject sequence. For the reads preliminarily assigned to the same species, a consensus sequence was generated using Medaka (v. 0.10.1). Then, the consensus sequence was remapped to the 16S rDNA/ITS reference database, and the best-assigned taxon was used as the final detection result of reads from the same species of the preliminary taxonomy assignment. The pathogen detection of the clinical sample was interpreted according to a strict set of rules as follows (M. Wang et al., unpub. data, https://doi.org/10.1101/2020.04.08.20057604). The criteria for calling a positive result of bacterial or fungal identification by NTS is as follows: mapped reads of bacterial species in specimens >100 or is more than that of any other species, and the ratio of mapped reads in the specimen and negative control >10; mapped reads of fungi from the species level

>20 or higher than 50% of relative richness, and the ratio of mapped reads in the specimen and negative control >10. There are critical lists of bacteria and fungi that have been clinically known to be typically or potentially pathogenic reported from clinical guidelines and literature (*9*). The coverage and proportion of *Rickettsia typhi* detected by NTS in bronchoalveolar lavage fluid are provided (Appendix Figure 1).

PCR and Sanger Validation

PCR reactions were performed in a 40 µL volume in a Thermal Cycler (Monad Biotech Co., Ltd, WuHan). The reaction mixture contained 20 µL 2× Rapid Taq Master Mix (Vazyme Biotech Co., Ltd, NanJing), 1.6 µL each of 10 µM primer, 11.8 µL water, and 5 µL of sample DNA. DNA was amplified using the following PCR profile: 3 min of denaturation at 95°C; followed by 38 cycles of 95°C for 15 s; annealing (60°C) for 15s; and 72°C for 5 s; with a final extension step at 72°C for 5 min. All primers were manufactured by Wuhan GeneCreate Biologic Engineering Co., Ltd (Appendix Table 1). The PCR products were analyzed by agarose gel electrophoresis and purified with a DNA gel extraction kit (Simgen). Sanger sequencing was performed on an ABI PRISM 3730 DNA Sequencer (Applied Biosystems, Foster City, CA, USA) for validation. Then, sequence information was aligned with database using NCBI BLAST (https://blast.ncbi.nlm.nih.gov/Blast.cgi) online software to make sure the NTS data were consistent with Sanger sequencing. Confirmatory PCR for *R. typhi* gene was applied for the detection of murine typhus in the BALF of this patient (Appendix Figure 2).

References

1. Huang Q, Fu A, Wang Y, Zhang J, Zhao W, Cheng Y. Microbiological diagnosis of endophthalmitis using nanopore targeted sequencing. Clin Exp Ophthalmol. 2021;49:1060–8. PubMed https://doi.org/10.1111/ceo.13992

2. Bursle E, Robson J. Non-culture methods for detecting infection. Aust Prescr. 2016;39:171–5. PubMed https://doi.org/10.18773/austprescr.2016.059

3. Childers R, Liotta B, Wang P, Katoula J, Thien T, Montilla-Guedez H, et al. 279 Overdiagnosis of urinary tract infections in the emergency department. Ann Emerg Med. 2021;78:S113. https://doi.org/10.1016/j.annemergmed.2021.09.292

4. Calus ST, Ijaz UZ, Pinto AJ. NanoAmpli-Seq: a workflow for amplicon sequencing for mixed microbial communities on the nanopore sequencing platform. Gigascience. 2018;7:giy140. PubMed https://doi.org/10.1093/gigascience/giy140

5. Fujita SI, Senda Y, Nakaguchi S, Hashimoto T. Multiplex PCR using internal transcribed spacer 1 and 2 regions for rapid detection and identification of yeast strains. J Clin Microbiol. 2001;39:3617–22. PubMed https://doi.org/10.1128/JCM.39.10.3617-3622.2001

6. Gohl DM, Vangay P, Garbe J, MacLean A, Hauge A, Becker A, et al. Systematic improvement of amplicon marker gene methods for increased accuracy in microbiome studies. Nat Biotechnol. 2016;34:942–9. PubMed https://doi.org/10.1038/nbt.3601

7. Kim B-J, Lee S-H, Lyu M-A, Kim S-J, Bai G-H, Kim S-J, et al. Identification of mycobacterial species by comparative sequence analysis of the RNA polymerase gene (rpoB). J Clin Microbiol. 1999;37:1714–20. PubMed https://doi.org/10.1128/JCM.37.6.1714-1720.1999

8. Benítez-Páez A, Portune KJ, Sanz Y. Species-level resolution of 16S rRNA gene amplicons sequenced through the MinION™ portable nanopore sequencer. Gigascience. 2016;5:4. PubMed https://doi.org/10.1186/s13742-016-0111-z

9. Miao Q, Ma Y, Wang Q, Pan J, Zhang Y, Jin W, et al. Microbiological diagnostic performance of metagenomic next-generation sequencing when applied to clinical practice. Clin Infect Dis. 2018;67(suppl_2):S231–40. PubMed https://doi.org/10.1093/cid/ciy693

Appendix Table 1. Primers for targeted amplification in nanopore targeted sequencing*

Marker genes	Primer name	Sequence, 5′ to 3′	Reference
16S rRNA	8F	Barcode-GGATCCAGACTTTGATYMTGG	M. Wang†
	27F	Barcode-AGRGTTYGATYMTGGCTCAG	1
	38F	Barcode-GGCTCAGRWYGAACGCTRG	M. Wang†
	1492R	Barcode-RGYTACCTTGTTACGACTT	1
	1495R	Barcode-TASRGYTACCTTGTTACGA	M. Wang†
ITS1/2	ITS1	Barcode-TCCGTAGGTGAACCTGCGG	2
	ITS1–2	Barcode-GTGAACCTGCGGAAGGATCAT	M. Wang†
	ITS4	Barcode-TCCTCCGCTTATTGATATGC	2
	ITS4–2	Barcode-TATGCTTAAGTTCAGCGGGT	M. Wang†
rpoB	MF	Barcode-CGACCACTTCGGCAACCG	3
	MR	Barcode-TCGATCGGGCACATCCGG	3
	MF-2	Barcode-GACGACATCGACCACTTCGG	M. Wang†
	MR-2	Barcode-GGGTCTCGATCGGGCACAT	M. Wang†

*ITS, internal transcribed spacer region.

†M. Wang et al., unpub. data, https://doi.org/10.1101/2020.04.08.20057604

Appendix Table 2. Primers for PCR tests used to detect *Rickettsia typhi* in a patient with murine typhus

Pathogen	Forward, 5′ to 3′	Reverse, 3′ to 5′
Rickettsia typhi	CATTTTTTATATAAAGGAAAAG	CTATTTCCATGCTGGGCTTACT

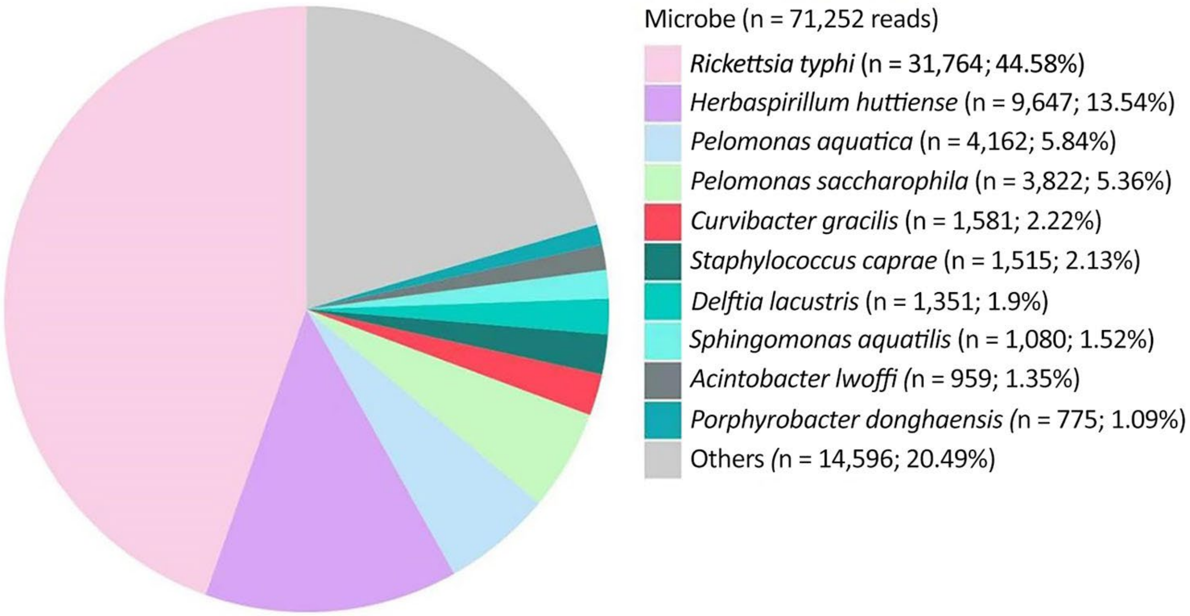

Appendix Figure 1. The coverage and proportion of *Rickettsia typhi* detected by nanopore targeted sequencing in bronchoalveolar lavage fluid of patient with diagnosed murine typhus, China.

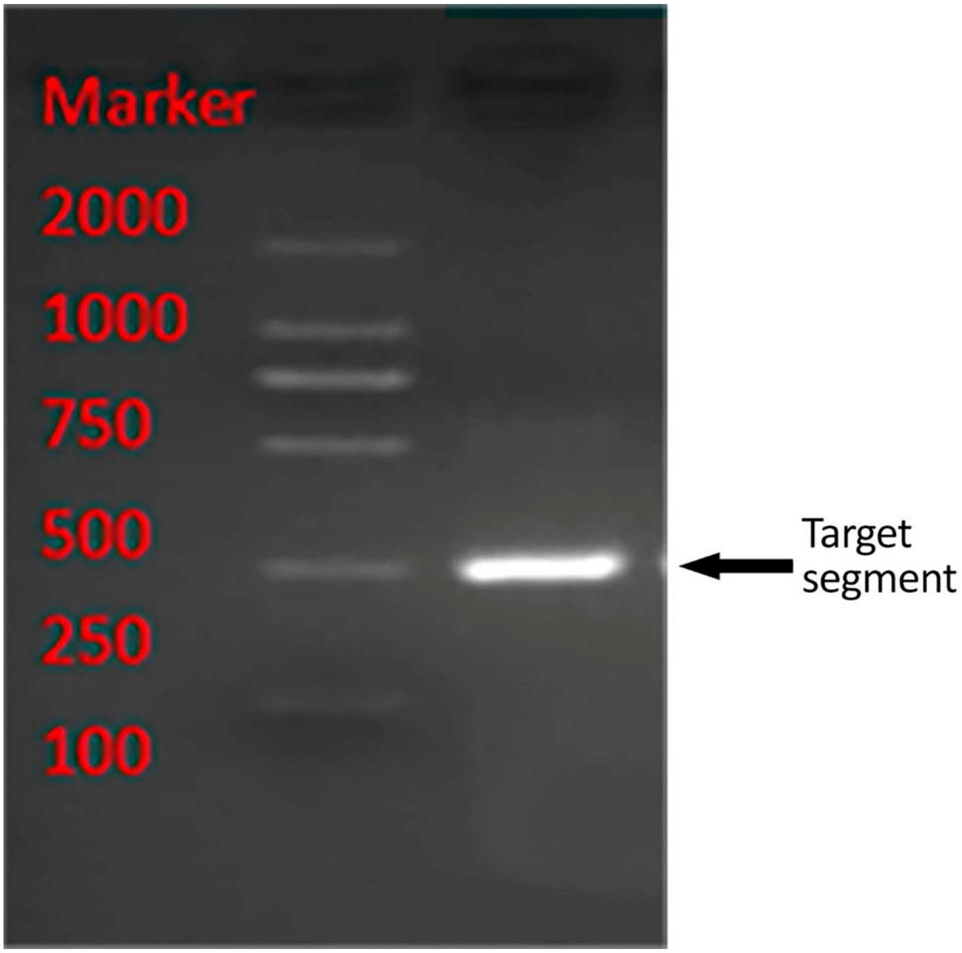

Appendix Figure 2. Confirmatory PCR for *R. typhi* gene in bronchoalveolar lavage fluid from a patient with diagnosed murine typhus, China.
